# Segmental schwannomatosis: characteristics in 12 patients

**DOI:** 10.1186/s13023-019-1176-4

**Published:** 2019-08-22

**Authors:** Abdulqader Alaidarous, Beatrice Parfait, Salah Ferkal, Joëlle Cohen, Pierre Wolkenstein, Juliette Mazereeuw-Hautier

**Affiliations:** 1 Service de Dermatologie, Hôpital Larrey, CHU de Toulouse, Toulouse, France; 2 Service de Génétique et Biologie Moléculaires, Hôpital Cochin, Assistance Publique-Hôpitaux de Paris, Paris, France; 3 UMR INSERM 1016 Institut Cochin & Université Paris Descartes, Sorbonne Paris Cité, Paris, France; 4 Service de Dermatologie, Hôpital Henri Mondor, Assistance Publique-Hôpitaux de Paris, Creteil, France

**Keywords:** Segmental schwannomatosis, Neurofibromatosis, LZTR1 mutation

## Abstract

**Background:**

Segmental schwannomatosis is characterized by multiple schwannomas affecting one-limb or less than 5 contiguous segments of spine. Its characteristics are not well described in the literature. Our objective was to better describe the demographic and clinical characteristics of this condition.

**Methods:**

This was a retrospective, bi-center study conducted in two French expert centers for neurofibromatosis and schwannomatosis. The clinical, radiographic, pathological and molecular aspects were extracted from patients’ clinical records.

**Results:**

Twelve patients with segmental schwannomatosis were identified. Eight were female and 4 were male. The median age at initial symptom was 29 years (range: 6–60 years) and the median age at diagnosis was 34.5 years (range: 13–65 years). Pain was the initial symptom for the majority of patients (7 of 12). The number of tumors was variable with six patients having more than 10 tumors. Peripheral distribution was seen in all patients. Quality of life could be impaired (median Dermatology Life Quality Index score was 4.5 (range: 2–13). The median duration of follow up was 3 years (range: 1–26). Chronic pain was the main complication (9 of 12 patients). Surgical intervention to control chronic pain was performed for 9 patients of whom 5 experienced recurrence of tumors. Molecular investigations revealed heterozygous *LZTR1* variants in 3 of 9 patients.

**Conclusion:**

Segmental schwannomatosis is a rare condition that may start early in life and often remains undiagnosed for many years. Pain is the main symptom and consequently could impair the quality of life. Surgery seems to be effective, but recurrences are frequent. Some patients carried heterozygous *LZTR1* variants. Further studies are needed to better understand this rare condition.

## Background

Schwannomas are rare benign tumors of the peripheral nerve sheaths that can appear sporadically as solitary lesions in the general population. When present in a multiple form, they are associated with neurofibromatosis type 2 (NF2) or schwannomatosis.

NF2 is an autosomal dominant condition caused by mutations in the *NF2* gene located on chromosome 22q12.2. Affected individuals inevitably develop schwannomas typically affecting both vestibular nerves. Other cerebral tumors can also occur during the course of the disease.

Whereas the hallmark of NF2 is the presence of bilateral vestibular schwannomas, schwannomatosis is characterized by the presence of multiple non-vestibular non-intradermic schwannomas. However, recent reports suggested that patient with schwannomatosis may also develop unilateral vestibular schwannoma or meningioma [1, 2].

A recent study was conducted in UK to describe the epidemiology of these two entities. According to the study, schwannomatosis has less than half the prevalence and birth incidence of NF2. Regional prevalence for schwannomatosis was 1 in 126,315 with a calculated birth incidence of 1 in 68,956 cases (prevalence of 1 in 50,500 and birth incidence of 1 in 27,956 for NF2). Life expectancy was significantly better in schwannomatosis compared to NF2 (mean age at death of 76.9 and 66.2, respectively) [3].

Diagnostic criteria have been developed to distinguish schwannomatosis from NF2. In 1996, MacCollin et al. reported 14 patients with multiple pathologically defined schwannomas without vestibular localization and developed the first research criteria for schwannomatosis [4]. In 2005, they proposed consensus diagnostic criteria for clinical use that were modified the following year by Baser and colleagues to augment their specificities [5, 6]. These modified diagnostic criteria suggest that patients with schwannomatosis must not fulfill any of the existing sets of diagnostic criteria for NF2, have no evidence of vestibular schwannoma on high-quality magnetic resonance imaging scan (MRI), no first-degree relative with NF2, and no known constitutional *NF2* mutation. A new approach was proposed in 2013 by Plotkin et colleagues who take into consideration the genetic mutation as a diagnostic criterion. In addition, they consider the diagnosis of schwannomatosis in those who had a unilateral vestibular schwannoma and also in patients with intracranial meningioma [7].

Several studies have been performed to identify the molecular aspect of schwannomatosis. In 1996, Jacoby et al. described two pathogenic mechanisms that cause schwannomatosis, including mosaic alteration at the *NF2* locus and somatic accumulation of *NF2*-gene mutations [8]. Subsequent, genetic studies showed that the *NF2* locus was excluded as the cause for familial schwannomatosis. Hulsebos et al. reported in 2007 a constitutional *SMARCB1 (*SWI/SNF related, matrix associated, actin dependent regulator of chromatin, subfamily b, member 1) mutation in a family affected by schwannomatosis [9]. Mutations in *SMARCB1* are estimated to occur in approximately 40–50% of familial cases and in 8–10% of sporadic cases [10]. In 2008, Sestini et al. reported a patient with *SMARCB1* mutations associated with somatic *NF2* mutations in schwannomatosis-associated tumors [11]. In 2014, a new gene: *LZTR1* (leucine zipper like transcription regulator 1) has been reported to be mutated in 80% of *SMARCB1* mutation-negative schwannomatosis patients with somatic molecular alterations of chromosome 22q including distinct different somatic *NF2* mutations as well as the loss of 22q in multiple tumors of a given patient. Subsequent studies performed with schwannomatosis patients with molecularly uncharacterized schwannomas reported mutation detection rates of 22–30 and 38% in sporadic and familial cases respectively [12–15].

All these genes, which are located on chromosome 22q, appear to act in regulating cell growth in the nervous system. They encode for proteins that act as tumor suppressor and control cell proliferation. Therefore, mutations of these genes may promote cells growth without control or order to form a tumor. However, current genetic testing does not reveal a mutation in all affected individuals, and there may be additional genes responsible for schwannomatosis yet to be discovered.

Segmental schwannomatosis (SS) is a limited form of schwannomatosis, defined by MacCollin as multiple schwannomas located on one limb or on five or less contiguous segments of spine [5]. The pathophysiology is unknown, and the characteristics of SS are not well described in the literature. We report here a series of SS in order to better describe their characteristics, including molecular aspects.

## Methods

### Patients

This was designed as a retrospective, bi-center study. The clinical records of all patients seen between June 2006 and June 2017 in two French expert centers for neurofibromatosis and schwannomatosis (Henri-Mondor Hospital in Paris and Larrey Hospital in Toulouse) were reviewed. All patients with a diagnosis of SS were included in the study. The definition of SS was based on the criteria by MacCollin and the modified diagnostic criteria of Baser [5, 6]. Histological analysis was obtained for all patients either after performing a biopsy as a diagnostic procedure or after a surgical intervention for painful lesions. Patients with incomplete data necessary for confirming the diagnosis (no histological analysis, no cerebral MRI and no MRI of the affected area) were excluded. Data were extracted from patients’ clinical records (age of onset, initial presenting symptoms, location of tumors, pain evaluation, ophthalmologic evaluation, quality of life (QOL), genetic data and family history).

Pain was evaluated by using the EVA scale (Pain Comparison Whit Visual Analog Scale: a subjective measure of patient’s pain intensity represented by a point between “0 = No pain at all” and “10 = worst pain imaginable”). QOL was assessed by using the Dermatology Life Quality Index (DLQI: a ten-question questionnaire used to measure the impact of skin disease on the quality of life of an affected person).

### Molecular investigations

After information on genetic testing, signed consent was obtained for 9 of the 12 patients. DNA was isolated from peripheral blood leucocytes using the Maxwell® 16 system and Maxwell® 16 LEV Blood DNA Kit (Promega). *NF2*, *SMARCB1* and *LZTR1* genes were investigated on the next-generation sequencing (NGS) facility of Cochin Hospital in Paris, France as already reported by Louvrier et al. [16] Briefly, a custom Ampliseq panel targeting the coding sequences of these genes was designed using the Ampliseq Designer plugin [17]. Preparation of NGS libraries, amplification, purification, emulsion PCR, enrichment, loading on Ion 316™ chips, sequencing with an Ion Personal Genome Machine® (PGM™) System (Thermo Fisher Scientific). Sequence alignment was performed with the Torrent Mapping Alignment Program (TMAP) [18]. Single nucleotide variants (SNVs) and short insertions and/or deletions detection from the bam files was performed using the Torrent Suite Variant Caller (TSVC) plugin from the Torrent Suite Software v5.0.4 [19]. Major calling parameters were chosen as follows to avoid any false negative result: minimum sequencing depth ≥ 5X for single nucleotides variants (SNVs), multiple nucleotide variants or complex variants and ≥ 10X for short insertions and/or deletions, minimum allele frequency (MAF) ≥ 1% for all using the TSVC. The 3’UTR region of *SMARCB1* was investigated by Sanger sequencing.

Copy number variations (CNVs) analysis was also performed by using quantitative values (number of reads for each amplicon of each sample) of the *Coverage Analysis* plugin on the Ion Torrent Browser 5.0.4.0 (Life Technologies) [16].

## Results

A total of 12 patients with SS were included. Their demographic and clinical characteristics are described in Table 1. These patients represented 28.6% of all patients diagnosed with schwannomatosis in the study period of more than 11 years (total of 48 patients: 28 with generalized form, 12 with segmental form and 2 with solitary lesion). Six patients were excluded due to incomplete data.

**Table 1:** Characteristics of patients

Patient	Sex	Age at the initial symptom (Y)	Age at Diagnosis (Y)	Family History	Initial Symptom	Number of tumors	Location of the tumors	Follow up (Y)	Pain (score before/after treatment)/10	DLQI Score/30	Treatment	Recurrence	Molecular investigation (*NF2, SMARCB1, LZTR1*)
1	M	53	54	–	P	3	UL	7	4/0	2	S	No	No mutation
2	F	ND	23	–	Mass	2	LL	1	ND	ND	S	Yes	*LZTR1* heterozygous (VAF of 47%) pathogenic variant c.692delT, p.(Phe231Serfs*21)
3	F	39	39	suspected NF1	P	> 10	LL	11	ND	ND	D (pregabaline) S	No	No mutation
4	F	6	38	suspected NF1	ED	9	UL	3	9/0	5	S	Yes	*LZTR1* heterozygous (VAF of 48%) VUS c.764 T > G, p.(Leu255Arg) (described in Louvrier et al. 2018)
5	M	ND	37	–	P	> 10	LL	2	ND	ND	S	No	No mutation
6	F	ND	55	–	ED	2	LL	1	ND	ND	S	No	NP
7	F	30	32	–	P	> 10	UL	26	8/2	ND	S	Yes	NP
8	M	12	13	–	P	> 10	UL	2	8/1	4	S	Yes	No mutation
9	M	15	18	Cerebral tumor	ED	5	UL	L	ND	ND	ND	ND	NP
10	F	29	32	–	P	> 10	LL	3	9/5	13	D (gabapentine, duloxetine, paracetamol)	NA	No mutation
11	F	19	28	Vestibular schwannoma	P	6	LL	3	6/0	7	D (pregabaline, tramadol) S	Yes	*LZTR1* heterozygous (VAF of 51%) mutation c.264-13G > A, p. p.Lys89Cysfs*16 (described in Piotrowski et al. 2014)
12	F	60	65	–	ED	> 10	LL	2	8/5	2	D (pregabaline)	NA	No mutation

*D* Drugs, *DLQI* Dermatology Quality of Life Index, *ED* electrical discharge, *F* female, *M* male, *L* Lost to follow-up, *LL* Lower limb, *NA* not applicable, *ND* not determined, *NF1* neurofibromatosis type 1, *NP* not performed, *P* Pain, *UL* Upper limb, *S* Surgery, *Y* year, *VAF* variant allele frequency, *VUS* variant of unknown significance

The majority of patients were female (8 patients: 67%). The median age at the initial symptom was 29 years (range: 6–60 years) and the median age at diagnosis was 34.5 years (range: 13–65 years). The median duration from the initial symptom to the diagnosis of SS was 3 years (range: 5 months-32 years). A family history of confirmed vestibular schwannoma was identified in one patient (grandfather of patient 11, no available details). Two other patients had grandparents with suspected but not proven neurofibromatosis type 1. One patient had a father who died from cerebral tumor of unknown origin.

The initial symptom was pain for 7 (58.3%) patients. For the remaining patients, four (33.3%) presented with numbness or electrical discharge sensation along the trajectory of the affected nerve and one (8.3%) patient presented with painless masses. Other clinical findings included café-au-lait macules in 3 (25%) patients (2 to 6 macules). Patients were otherwise healthy.

Half of the patients had multiple (more than 10) tumors. The remaining patients had 2 to 9 tumors.

SS had a peripheral distribution in all patients (lower limbs location in 7 patients).

A clinical ophthalmologic evaluation was performed for all patients to exclude any stigmata of NF2. All patients had normal evaluation.

One patient was lost to follow up and for the remaining 11 patients, the median duration of follow up was 3 years (range: 1–26 years).

SS was complicated by chronic pain in 9 (75%) patients. Seven patients presented initially with pain while electrical discharge was the initial symptom for the other two patients. This pain was either localized to the tumor, radiating along the nerve of origin or felt in areas where there are no adjacent tumors. The pain became intense gradually over time and ranges in intensity from mild to severe. An evaluation of the pain according to EVA scale was available for 7 of the 12 patients. The median score was 8 (range 4–9/10) before any medical or surgical intervention.

Quality of life was assessed in 6 patients using the Dermatology Life Quality Index. The median score was 4.5 (range: 2–13). Only one patient could be considered having an impaired QOL since the score was more than 10. Impairment was related to difficulties in daily activity ranging from mild to moderate disability especially if activity depends on the utility of the affected region. For example, 3 patients with affected hand reported a difficulty during working, writing, sports and manual gesture. This difficulty has been adapted with time and patients became more accommodated to their disease. None reported feeling depressed because of their disease at the time of the study and none used antidepressants. None took day-off from work because of incapability.

Surgical intervention of schwannomas was performed in 9 (75%) patients to alleviate their chronic pain. Recurrence occurred in 5 patients, who needed repeated surgery. Efficacy of the surgery, in term of pain management, was evaluated using EVA scale for 5 patients. The pain disappeared in 3 patients (score equal to zero) and decreased to a low score of 1 or 2/10 for the remaining 2 patients. Medications were introduced in 4 patients: 2 patients (patients 3 and 11) had been treated before the surgical intervention without any notable improvement, 1 patient (patient 12) refused the surgery and in 1 patient (patient 10), the surgery was inapplicable. The main analgesics used were paracetamol, opioid drugs or neuropathic agents such as gabapentin, pregabaline and duloxetine. The efficacy of oral medication was only assessed in 2 patients, pain was reduced but the score remained at 5/10.

Molecular analysis (*NF2*, *SMARCB1* and *LZTR1)* was performed for 9 (75%) of the 12 patients. We did not identify any sequence variation in the coding sequences and exon/intron boundaries of *NF2* and *SMARCB1* genes with an allele frequency threshold of 1% for SNV and 20% for CNV. *SMARCB1* 3’UTR variants (including the recurrent c.*82C > T pathogenic variant) were also excluded by Sanger sequencing. *LZTR1* heterozygous variants were found in three patients. Patient 2 harbored the c.692delT pathogenic variant in exon 8 corresponding to the predicted deleterious p.(Phe231Serfs*21). Patient 4 harbored c.764 T > G variant in exon 8 corresponding to the predicted missense p.(Leu255Arg). This patient’s variant was already reported [16]. This variant was not described in population databases and was predicted deleterious by several prediction softwares leading to a variant of unknown significance classification according to the American College of Medical Genetics recommendations [20]. Patient 11 carried the heterozygous pathogenic transition c.264-13G > A corresponding to a splicing abnormality c.Lys89Cysfs*29 [12]. These 3 patients had multiple lesions localized either on upper or lower limb.

## Discussion

We report here a series of SS. This series shows that SS is a rare condition (12 cases over a period of 11 years in 2 expert academic centers) that may start early in life and often remains undiagnosed for many years. The number of lesions is variable, and SS often has a peripheral distribution. Pain is the main symptom and QOL could be impaired. Three patients carried *LZTR1* mutations.

There are some limitations to our study, related to its retrospective nature. Some patients were excluded for missing data, the duration of follow-up was limited, and one patient was lost to follow-up. Therefore, the possibility of evolution towards generalized schwannomatosis or even NF2 cannot be excluded for all patients. Another limitation is the absence of whole-body MRI scanning that could have discovered asymptomatic schwannomas out of the segmental area. Finally, molecular analysis was performed for only 9 of the 12 patients and due to the unavailability of two distinct tumor specimens, we could not perform molecular analysis to exclude the presence of mosaicism in tissues.

Our review of the literature revealed only 2 small series of 5 and 6 patients with SS from the same Chinese center in 2013. These series described the clinical, histological and radiological aspects but not the molecular features [21, 22]. (Table 2).

**Table 2:** Characteristics of the patients with segmental schwannomatosis reported in the 2 series of the literature

Study	Patients with SS: n (%) / total schwannomatosis	Sex (M/F)	Mean age at onset: Y (range)	Mean age at diagnosis: Y (range)	Family History (n)	Symptoms: n (%)	Localization	Surgery	Follow up (range)
Wang [21]	5 (1.42%) / 351	1/4	ND	38 (29–48)	None	P: 3 (60%) N: 2 (40%)	UL	+	4.5 Y (5 months- 11 Y)
Chen [22]	6 (75%) / 8	1/5	30.3 (24–41)	34.5 (24–54)	Suspected NF1	P: 3 (37.5%) Mass: 1 (12.5%) P & Mass: 1 (12.5%) N & Mass: 1 (12.5%%)	UL	+	28.5 months (8–50 months)

*F* female, *M* male, n: number, *N* numbness, *ND* not determined, *NF1* neurofibromatosis type 1, *P* pain, *SS* segmental schwannomatosis, *UL* Upper limb, *Y* year

In a retrospective analysis of 87 patients with schwannomatosis, 26 had a segmental form. The only reported characteristic was the location: involvement of the leg (35%), arm (23%), spine (23%) or other locations (19%) [23]. We also found few case reports describing this clinical entity and two studies reporting the molecular analysis without details about their clinical features [24].

With regards to the frequency of SS, it represented nearly a third of all patients with schwannomas in our study, similarly to the series by Merker [23]. It rose up to 75% in the study conducted by Chen et al. and was much lower in the other series by Wang et al. (1.4%) [21, 22]. Our series showed a female predominance, similarly to these 2 other series. In contrast, no female predominance has been reported for the classic form of schwannomatosis.

In our study, the age at the initial symptom was in accordance with the observation of Chen et al. (29 vs. 30.3 years). Similarly, the median age at diagnosis in our series was in accordance with Chen and Wang studies (34.5 vs 34.5 and 38 years, respectively) [21, 22].

The majority of our cases presented with pain (58.3%), similarly to the 2 other series (50 and 60%) [21, 22]. The peripheral distribution of the tumors is a common feature between our series and the 2 series of the literature. Nevertheless, all patients in the other series had only an involvement of the upper extremities, contrarily to our patients.

Surgical resection of tumors seems to be effective on pain control. This outcome is similar to what was observed in the other series. However, some locations were not accessible to surgery and then needed other treatment modalities. The percentage of recurrence in our series was much higher than the 2 other series (55.6% vs. 16.7% or 20%). Medical treatment is another option. It was considered by our patients as not effective. It was not evaluated in the other series.

QOL was not previously assessed in the literature. Our series showed that SS may affect QOL. We can hypothesize that pain contributes to this impaired QOL.

The pathogenesis of the SS remains unclear. Somatic mosaicism was suggested as the underlying cause by Leverkus who reported one patient with SS presenting with multiples lesions on the left forearm. Biopsy specimens of two different lesions showed two distinct mutations of the *NF2* gene, with concomitant loss of heterozygosity in both tumors thus excluding a *NF2* mosaic event [24]. Farschtschi studied 5 patients with SS, using magnetic resonance neurography and mutation analysis of *NF2*, *SMARCB1*, and *LZTR1*. In 4 of the 5 patients, subtle fascicular nerve lesions were detected in clinically unaffected extremities. Two patients exhibited *LZTR1* germline mutations. This appears contrary to a simple concept of somatic mosaicism and suggests more complex and heterogeneous mechanisms underlying the phenotype of SS than previously thought [25].

In our series, three patients harbored a heterozygous (possibly germline) *LZTR1* variant on peripheral blood confirming that the genetic changes causing clinically defined segmental schwannomatosis include *LZTR1* gene alterations. The mutation c.264-13G > A in intron 2 has been previously reported by Piotrowski et colleagues in a patient presenting with spinal schwannomas and schwannomas on forearm and abdomen. The patient carrying c.764 T > G variant of unknown significance was already reported by Louvrier, and the c.692delT pathogenic variant has not been previously reported in the literature.

One of the main challenges in diagnosing SS is to differentiate it phenotypically from mosaic NF2 or early NF2 since the latter two can fulfill the current criteria of schwannomatosis [26]. Mosaic NF2 can present in the same phenotype as segmental schwannomatosis without vestibular tumor. According to Baser modified criteria, SS is excluded if a constitutional *NF2* mutation is found. In this context, we carefully analyzed the NGS data in order to identify mosaic events. We excluded a mosaic event in blood in *NF2*, *SMARCB1* and *LZTR1* for 6 out of 9 tested patients with a threshold of 1% for SNV and 20% for CNV. This does not exclude the presence of mosaicism in tissues not evaluated in our study or presence at levels below the sensitivity of our method. Further studies on two independent tumors would help to identify the molecular basis for the six *LZTR1*-negative patients. Together with literature results, our study shows the role of *LZTR1* loss of function in the SS phenotype for at least one third of the patients [25].

## Conclusions

In conclusion, SS is a rare and sometimes disabling disease that may start early in life and often remains undiagnosed for many years. Its pathogenesis is still unclear. By describing the characteristics of SS in 12 patients, our series contributes to expand knowledge of disease features. Additional studies are needed to better understand the pathogenesis and help to improve the management of these patients.

## Data Availability

All data generated or analyzed during this study are included in this published article and its supplementary information files.

## Abbreviations

CNVs: Copy number variations
DLQI: Dermatology Life Quality Index
DNA: Deoxyribonucleic acid
EVA: Pain Comparison Whit Visual Analog Scale
*LZTR1*: Leucine zipper like transcription regulator 1
MAF: Minimum allele frequency
MRI: Magnetic resonance imaging
NF2: Neurofibromatosis type 2
NGS: Next-generation sequencing
PCR: Polymerase chain reaction
PGM: Personal Genome Machine
QOL: Quality of life
*SMARCB1*: SWI/SNF related, matrix associated, actin dependent regulator of chromatin, subfamily b, member 1
SNVs: Single nucleotide variants
SS: Segmental schwannomatosis
TMAP: Torrent Mapping Alignment Program
TSVC: Torrent Suite Variant Caller
